# Extracellular matrix protein Reelin promotes myeloma progression by facilitating tumor cell proliferation and glycolysis

**DOI:** 10.1038/srep45305

**Published:** 2017-03-27

**Authors:** Xiaodan Qin, Liang Lin, Li Cao, Xinwei Zhang, Xiao Song, Jie Hao, Yan Zhang, Risheng Wei, Xiaojun Huang, Jin Lu, Qing Ge

**Affiliations:** 1 Key Laboratory of Medical Immunology, Ministry of Health, Department of Immunology, School of Basic Medical Sciences, Peking University Health Science Center, 38 Xue Yuan Road, Beijing, 100191, China; 2 Department of Biophysics, School of Basic Medical Sciences, Peking University Health Science Center, Beijing, 100191, China; 3 Peking University Institute of Hematology, People’s Hospital, Beijing, 100044, China

## Abstract

Reelin is an extracellular matrix protein that is crucial for neuron migration, adhesion, and positioning. We examined the expression of Reelin in a large cohort of multiple myeloma patients recorded in Gene Expression Omnibus (GEO) database and used over-expression and siRNA knockdown of Reelin to investigate the role of Reelin in myeloma cell growth. We find that Reelin expression is negatively associated with myeloma prognosis. Reelin promotes myeloma cell proliferation *in vitro* as well as *in vivo*. The Warburg effect, evidenced by increased glucose uptake and lactate production, is also enhanced in Reelin-expressing cells. The activation of FAK/Syk/Akt/mTOR and STAT3 pathways contributes to Reelin-induced cancer cell growth and metabolic reprogramming. Our findings further reveal that activated Akt and STAT3 pathways induce the upregulation of HIF1α and its downstream targets (LDHA and PDK1), leading to increased glycolysis in myeloma cells. Together, our results demonstrate the critical contributions of Reelin to myeloma growth and metabolism. It presents an opportunity for myeloma therapeutic intervention by inhibiting Reelin and its signaling pathways.

Multiple Myeloma (MM) accounts for 1% of neoplastic disease and 13% of hematological cancers. It is characterized by a malignant proliferation of plasma cells within the bone marrow (BM). Accumulation of genetic and epigenetic alterations in plasma cells and the complex interactions of plasma cells with cellular and non-cellular components of BM microenvironment promote neoplastic cell growth and drug resistance, making MM an incurable disease despite the recent improvements in the development of anti-tumor therapeutics^1^,^2^,^3^.

Reelin (*RELN*) is a large extracellular matrix glycoprotein that is crucial for neuron migration, adhesion, and positioning. Binding of Reelin to its receptors, including ApoER2 and VLDLR, induces Src family kinase-mediated phosphorylation of the intracellular adaptor protein disable-1 (Dab1). It subsequently leads to the activation of multiple signaling pathways that orchestrates cortex lamination during embryonic brain development^4^,^5^.

Several types of cancer cells upregulate Reelin expression, including multiple myeloma, high Gleason score prostate cancer, esophageal carcinoma, and retinoblastoma^6^,^7^,^8^,^9^,^10^. Reelin is found to suppress the migration of transforming growth factor β1-induced esophageal carcinoma cell migration while promoting the adhesion and survival of multiple myeloma cells^6^,^10^. The activation of VLDLR/Dab1-independent integrin β1 signaling pathway is involved in Reelin-mediated myeloma cell adhesion and survival^10^. As multiple studies have indicated that adhesion of myeloma cells to BM microenvironment leads to enhanced tumor cell growth^11^,^12^, it is thus important to learn whether Reelin plays a role in myeloma cell proliferation. In addition, enhanced aerobic glycolysis (the Warburg effect) has been well considered as one of the fundamental metabolic alterations that gives survival and growth advantages to tumor cells^13^,^14^. Whether integrin signaling pathway activated by Reelin is associated with metabolic reprogramming in myeloma cells is not known. Here we show for the first time that Reelin plays an important role in the regulation of myeloma cell proliferation and glucose metabolism. We further confirm the expression of Reelin in a large cohort of MM patients recorded in Gene Expression Omnibus (GEO) database and demonstrate its negative association with prognosis.

## Results

### High RELN expression correlates with poor event-free survival and overall survival in MM patients

**Figure 1:**
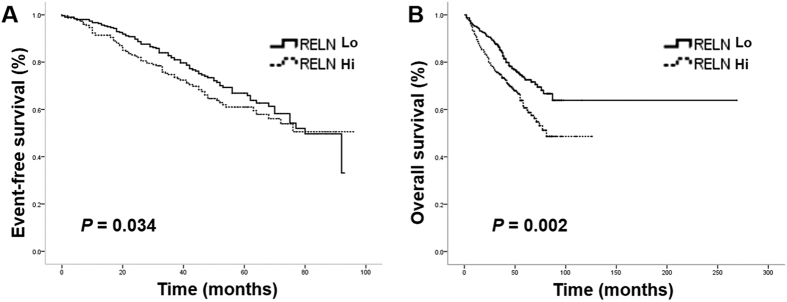
*RELN* expression is negatively associated with EFS and OS in multiple myeloma patients. The expression levels of RELN from 565 newly diagnosed MM patients from GSE24080 were first transformed by log-base 2 and were then analyzed by a hierarchical cluster analysis with Ward’s method. The cut-off value (810) was defined. The Kaplan-Meier method was used to plot the event-free survival (EFS) (**A**) and overall survival (OS) (**B**), which were compared between patients with high and low *RELN* expression using the log-rank test.

We previously found negative association of *RELN* expression with progression-free survival (PFS) and overall survival (OS) in 70 MM patients in China^10^. To confirm the relationship between *RELN* expression profile and matched clinical information in a larger cohort of patients with multiple myeloma, a publicly available Gene Expression Omnibus (GEO) database, including 565 newly diagnosed MM patients (USA) from GSE24080 (Affymetrix HG-U133_Plus_2.0 array) (www.ncbi.nlm.nih.gov/geo/query/acc.cgi?acc=GSE24080) was analyzed^15^. A hierarchical cluster analysis with Ward’s method was first performed to analyze the expression level of *RELN* (the probe set 205923_at) in these patients from GSE24080. A cut-off value was then set at 810-relative expression unit to separate low from high *RELN* expression. The group with low *RELN* expression had better event-free survival (EFS) and OS than that with high *RELN* expression (Fig. 1A,B). The Median EFS for low and high *RELN* expression groups were 44 months (95% confidence interval (CI): 41.3, 46.1) and 40 months (95% CI: 37.1, 43.0), respectively (*P* = 0.034). The OS for low and high *RELN* groups were 52 months (95% CI: 49.2, 54.9) and 47 months (95% CI: 44.3, 50.5), respectively (*P* = 0.002). In addition, high *RELN* expression was associated with more focal lesions defined by Magnetic Resonance Imaging (MRI) and higher levels of lactate dehydrogenase (LDH) (*P* < 0.001, *P* = 0.023, respectively) (sTable 1). We further performed multivariate analysis and found that *RELN* expression was an independent prognostic factor for OS (*P* = 0.041). These results implicate that Reelin may be involved in promoting MM cell growth and tissue damage in the bone marrow.

### Reelin promotes MM cell proliferation *in vitro*

**Figure 2:**
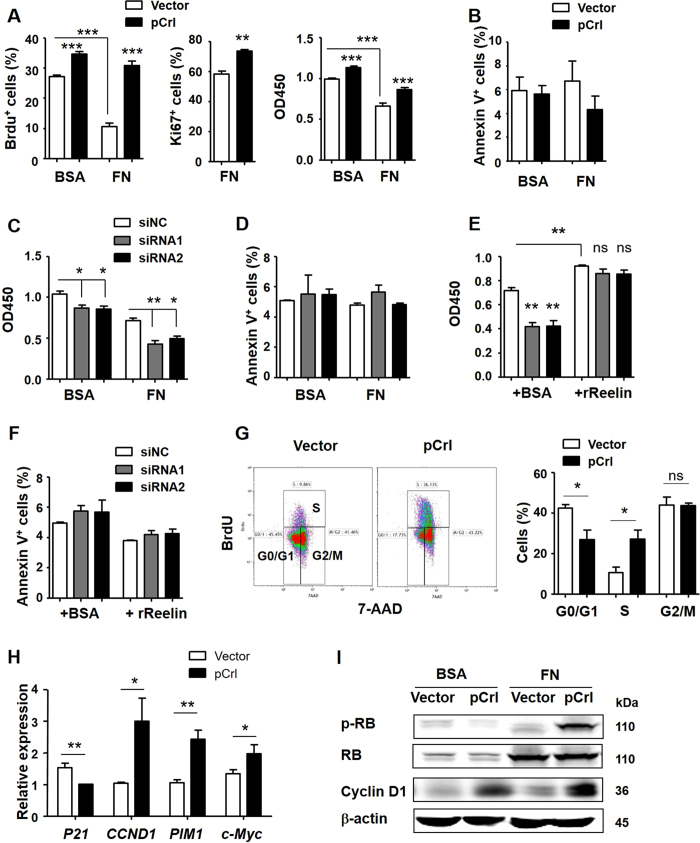
Reelin promotes MM cell proliferation *in vitro*. (**A**) Reelin over-expression promotes H929 cell proliferation in the presence or absence of fibronectin. H929 cells were transfected with 10 μg pCrl or control vector for 40 hours. The cells were then seeded in 5% BSA- or 40 μg/ml FN-coated plates in the absence of cytotoxic agents. Twenty-four hours later, the cell proliferation was analyzed by BrdU, Ki67 and CCK8 method staining. (**B**) Reelin over-expression does not alter cell survival in the absence of chemotherapeutic agents. H929 cells were treated as (**A**). The cell apoptosis was analyzed by annexin V staining. (**C,D**) Reelin knockdown by specific siRNAs suppresses H929 cell proliferation. H929 cells were transfected with 300 pmol Reelin specific siRNA or control siRNA (siNC) for 40 hours. The cells were then seeded in BSA- or FN-coated plates. The cell growth (**C**) and apoptosis (**D**) were analyzed 24 hours later by CCK8 method and annexin V staining, respectively. (**E,F**) The addition of Reelin reverses cell growth inhibition induced by Reelin-specific siRNAs. H929 cells were transfected with Reelin specific siRNAs or control siRNA. The cells were then seeded in FN-coated plates with recombinant Reelin (rReelin) or BSA control. The cell proliferation (**E**) and apoptosis (**F**) were analyzed 24 hours later. (**G**) Reelin promotes myeloma cell cycle progression from G1 to S phase. Reelin-overexpressing H929 cells were seeded in FN-coated plates for 24 hours. The cell cycle analysis by flow cytometry was then performed. (**H**) The transfection of H929 cells with pCrl alters the transcription of proliferation-related genes. (**I**) Reelin over-expression increased the levels of phosphorylated Rb and total Cyclin D1. The cells in (**A**) were harvested and cell lysates were subjected to western blotting with phospho-Rb (Ser780), Rb, and Cyclin D1-specific antibodies. An antibody specific for GAPDH was used as a loading control. The experiments were performed for 3 times and similar results were obtained. Error bars indicate the standard deviation. The data were evaluated by two-tailed Student’s *t*-test. *p < 0.05, **p < 0.01, ***p < 0.005.

To examine the impact of Reelin on MM cell proliferation, two human myeloma cell lines (HMCLs), NCI-H929 (shown as H929) and U266 cells were used. The cells were transfected with the plasmid pCrl to overexpress Reelin or Reelin-specific siRNAs to knock down intrinsic Reelin expression (sFig. 1). Compared to control vector (pcDNA3)-transfected H929 cells, pCrl-transfected ones in the presence or absence of fibronectin (FN) revealed higher level of proliferation measured by BrdU and Ki67 staining, and Cell Counting Kit-8 (CCK8), (Fig. 2A and sFig. 2A,B). This enhanced proliferation in Reelin-overexpressing cells is not due to different cell survival capability. In the absence of cytotoxic agents, the apoptosis was similar between cells transfected with pCrl and pcDNA3, or between cells seeded in BSA- and FN-coated wells (Fig. 2B). Notably, a significant cell growth inhibition was found in H929 cells seeded in FN-coated wells when compared to BSA-coated ones (Fig. 2A). This is in agreement with multiple reports suggesting that activation of β1 integrins (receptor for FN) in hematopoietic cells, including myeloma cells, induces a growth arrest^16^,^17^,^18^,^19^,^20^. However, the overexpression of Reelin alleviated the adhesion-induced growth inhibition (Fig. 2A). Similar increase in cell proliferation was found in Reelin-overexpressing U266 cells (sFig. 2C).

When examining HMCLs transfected with Reelin-specific siRNAs, the proliferation of these cells in either BSA- or FN-coated wells were significantly lower than that of cells transfected with the control siRNA (Fig. 2C). Again, the cell survival was similar in these myeloma cells (Fig. 2D). We further added recombinant Reelin (rReelin) protein to siRNA-transfected cells. The addition of Reelin not only promoted the proliferation of control siRNA-transfected cells, but also alleviated cell growth inhibition mediated by Reelin-specific siRNAs (Fig. 2E and sFig. 2D). The cell survival was not altered by rReelin in the absence of cytotoxic drug (Fig. 2F). These results indicate that Reelin specifically facilitates myeloma cell growth.

We also performed the cell cycle analysis. A decrease in G0/G1 phase and an increase in S-phase were found in Reelin-overexpressing cells, suggesting that Reelin promotes myeloma cell cycle progression from G1 to S phase (Fig. 2G). In line with the cell cycle changes, HMCLs with Reelin overexpression had significantly higher transcription levels of cell cycle promoting genes such as CCND1, PIM1, and c-Myc and lower level of cell cycle inhibitor p21 when compared to the control cells (Fig. 2H and sFig. 2E). The protein level of Cyclin D1 was higher in pCrl-transfected cells than the controls in the absence of FN. The cell adhesion to FN further increased Cyclin D1 in pCrl-transfected cells (Fig. 2I). Retinoblastoma (Rb) protein can be phosphorylated by the interaction of Cyclin D1 with Cdk4 and Cdk6, leading to the release of E2F from Rb and the activation of E2F target genes essential for DNA replication^21^,^22^. In FN-coated plates, the increased phosphorylation of Rb was only seen in pCrl-transfected H929 cells even though upregulated total Rb protein could be found in adherent cells transfected with pCrl and vector control (Fig. 2I). Thus, the decrease in p21, increase in Cyclin D1, and increase in Rb phosphorylation all indicate that Reelin facilitates myeloma cell proliferation by regulating key proteins involved in G1/S transition.

### Reelin promotes glycolysis in myeloma cells

**Figure 3:**
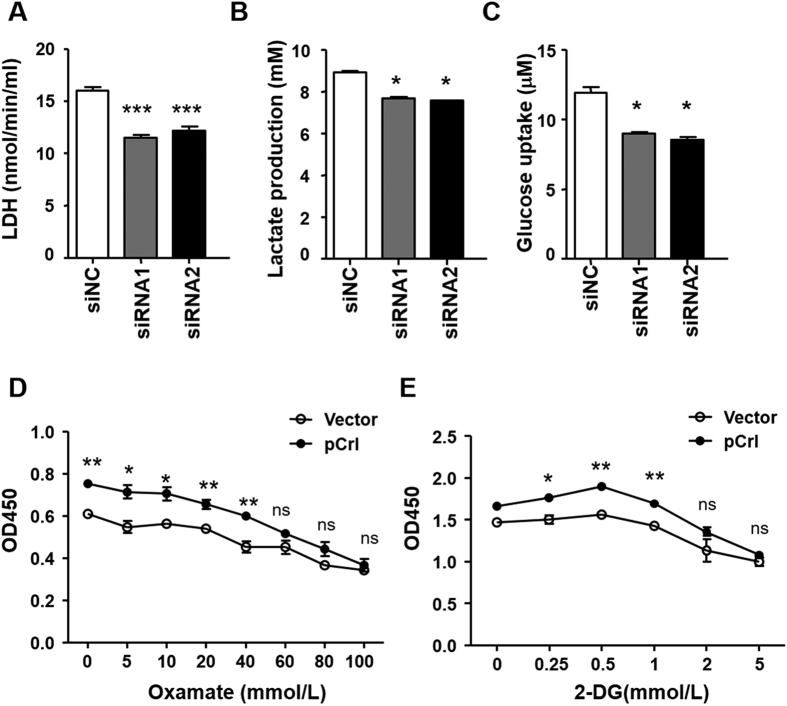
Reelin promotes myeloma cell glycolysis. (**A–C**) Reelin knockdown suppresses H929 cell glycolysis. H929 cells with Reelin-specific siRNAs or control siRNA for 40 hours. The levels of LDH (**A**), L-Lactate (**B**) and glucose uptake (**C**) were then measured. (**D,E**) Reelin over-expression alters the response of H929 cells to glycolysis inhibitors. H929 cells were transfected with pCrl or vector for 40 hours. The cells were then seeded in FN-coated plates with different concentrations of oxamate (0–100 mmol/L) (**D**) or 2-deoxy-D-glucose (2-DG) (0–5 mmol/L) (**E**). The cells were analyzed by CCK8 method 24 hours later.

Enhanced aerobic glycolysis, also known as the Warburg effect, gives tumor cell advantage for proliferation^14^. As the above CCK8 results indicate high dehydrogenase activities in Reelin-overexpressing cells and high Reelin expression is associated with high LDH level in MM patients, we determined whether Reelin is involved in the regulation of glucose metabolism in myeloma cells. Compared to controls, H929 with Reelin knockdown revealed decreased LDH level, lactate production, and glucose uptake in normoxia condition (Fig. 3A–C). We then tested whether Reelin overexpression could alter the cell response to glycolysis inhibition. A specific glycolysis inhibitor oxamate was chosen as it is a pyruvate analog that directly inhibits the conversion of pyruvate to lactate by lactate dehydrogenase. As shown in Fig. 3D, oxamate significantly inhibited MM growth in a dose-dependent manner. The cell survival was not affected within 100 mmol/L of oxamate (data not shown). At high concentration (60–100 mmol/L) of oxamate, the growth differences between pCrl- and vector-transfected cells became much less significant, suggesting that Reelin^hi^ cells are more sensitive to glycolysis inhibition. Similar results were obtained using another glycolysis inhibitor, 2-dexoy-D-Glucose (2-DG) (Fig. 3E). Together, these results indicate that Reelin-mediated cell proliferation is at least partially due to its role in promoting glycolysis in myeloma cells.

### Reelin promotes MM cell growth *in vivo*

**Figure 4:**
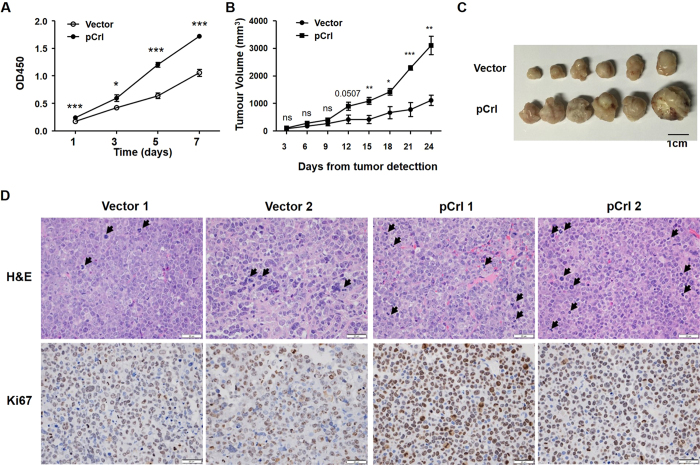
Reelin promotes MM cell growth *in vivo*. (**A**) Reelin-over-expressing H929 cell lines proliferate faster than the controls. H929 cell lines with stable transfection of pCrl or control vector were seeded in FN-coated plates. The cell growth was analyzed by CCK8 method at 1, 3, 5, and 7 days later. A statistical significance (*P* < 0.0001) was found by the univariate analysis with Tukey post-hoc tests. The cell growth differences at the same time point were compared by Student’s *t-*test. (**B,C**) Reelin promotes MM tumor growth *in vivo*. Eight-week old female NOD/SCID mice were subcutaneously inoculated with vector- or pCrl-transfected H929 cell lines (1 × 10^7^) in 100 μL of serum-free RPMI-1640. Each group had 6 mice. The tumor size was measured in two perpendicular dimensions once every 3 days after palpable tumors developed (left panel). The mice were sacrificed at day 24 and the picture of tumors was taken (right panel). The Tukey post-hoc test was used to analyze the statistical significance between the two groups (*P* < 0.0001). The tumor size differences at the same time point were compared by Student’s *t-*test. (**D**) Reelin promotes MM cell proliferation *in vivo*. The tumor sections were evaluated by H&E and Ki67 staining. Arrowheads represent cells in cell cycle. Two representative tumor images from each group at 20x magnification were shown. The experiment was repeated twice and similar results were obtained.

To examine whether Reelin facilitates MM growth *in vivo*, we prepared a Reelin-overexpressing H929 cell line and a vector control cell line. Similar to the result of transient transfection, Reelin-overexpressing cell line exhibited enhanced cell growth in tissue culture (Fig. 4A). When introduced into SCID/NOD mice, the growth of Reelin-overexpressing tumors were significantly faster than those of control tumors, resulting in larger tumors in the former group (Fig. 4B,C). The histological sections of Reelin-overexpressing tumors revealed higher numbers of dividing cells and higher percentages of Ki67^+^ cells (Fig. 4D). These results well support our *in vitro* findings of Reelin in promoting myeloma cell growth.

### Reelin promotes MM cell growth via FAK/Syk/STAT3 and Akt pathways

**Figure 5:**
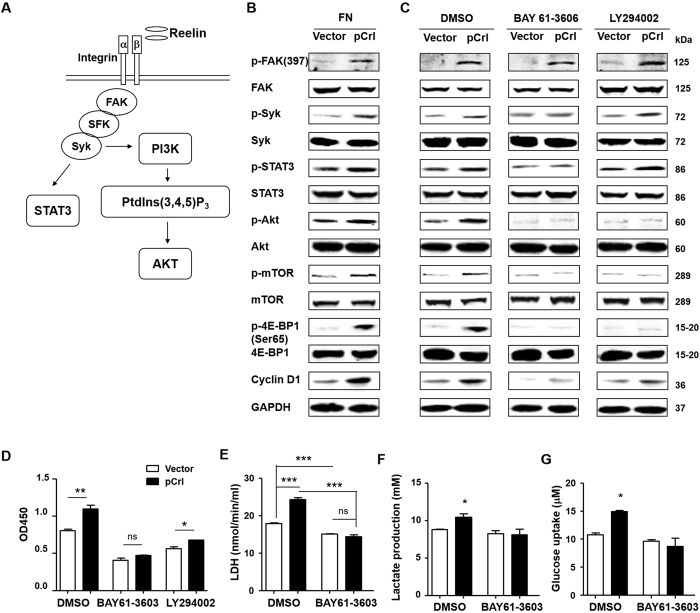
Reelin promotes MM cell growth and glycolysis via Syk/Akt pathway. (**A**) Diagram of Reelin-induced signaling pathways in MM cells. (**B**) Reelin induces the activation of Syk and Akt/mTOR pathways. H929 cells were transfected with pCrl or vector for 40 hours. The cells were then seeded in FN-coated plates. One hour later, the cells were harvested and cell lysates were subjected to western blotting with phospho-FAK (Tyr397), FAK, phospho-STAT3 (Tyr705), STAT3, phospho-Syk (Tyr525/526), Syk, phospho-Akt (Ser473), Akt, phospho-mTOR (Ser2448), mTOR, phospho-4E-BP1 (Ser65), 4E-BP1, and Cyclin D1-specific antibodies. An antibody specific for GAPDH was used as the loading control. (**C,D**) The suppression effect of Syk and PI3K inhibitors on Reelin-mediated cell growth. H929 cells were transfected with pCrl or control plasmid and were then cultured in FN-coated plates. The cells were treated with DMSO, Syk inhibitor BAY 61–3606, or PI3K inhibitor LY 294002 for 24 hours. A fraction of the cells were lysed and subjected to western blotting (**C**) and the rest were measured by CCK8 method (**D**). (**E–G**) Syk is involved in Reelin-induced glycolysis. H929 cells were transfected with pCrl or vector for 40 hours. The cells were then cultured in FN-coated plates and were treated with DMSO or Syk inhibitor BAY 61-3606 for 24 hours. The levels of LDH (**E**), L-Lactate (**F**) and glucose uptake (**G**) of the cells were then measured. The results are representative of two to three independent experiments.

We previously found that Reelin activates integrin β1 and promotes myeloma cell adhesion and survival via FAK/Src/Syk/STAT3 and Akt pathways (Fig. 5A)^10^. To examine whether the same pathways were involved in cell proliferation, we extracted proteins from FN-adherent cells transfected with pCrl or control vector and performed Western blotting. Compared to the controls, pCrl-transfected HMCLs revealed higher phosphorylation levels of FAK, Syk, STAT3, and Akt (Fig. 5B and sFig. 3A). The total amount of these signaling proteins did not appear to change. The activation of phosphoinositide 3-kinase (PI3K)/Akt can lead to the phosphorylation of the mechanistic target of rapamycin (mTOR) and the subsequent 4E-BPs (inactivation), allowing eIF4F complex formation and protein translation progression, a key process involved in cell growth and proliferation^23^,^24^. As shown in Fig. 5B and sFig. 3A, the phosphorylation of mTOR and 4E-BP1 was increased in Reelin-overexpressing cells while the total amount of proteins was similar. These data suggest that FAK/Syk/STAT3 and Akt/mTOR pathways activated by Reelin may be involved in facilitating myeloma cell growth.

To confirm the role of these signaling pathways on Reelin-mediated MM proliferation, we treated pCrl/control vector-transfected cells with Syk inhibitor BAY 61–3606. The inhibition of Syk in H929 cells suppressed the phosphorylation of STAT3, Akt, mTOR, and 4E-BP1 (Fig. 5C). BAY61–3606 treatment also inhibits Cyclin D1 upregulation in cells with pCrl (Fig. 5C). FAK phosphorylation was not affected. The growth difference between pCrl- and vector-transfected H929 cells was also diminished by Syk inhibition (Fig. 5D). Similar results were found in BAY61-3606-treated U266 cells ( sFig. 3B,C), indicating that Syk is critical for Reelin-mediated myeloma cell proliferation.

We further tested whether Syk is involved in Reelin-mediated metabolic reprogramming. Compared to the controls, H929 cells transfected with pCrl revealed elevated LDH and lactate levels, and enhanced glucose uptake (Fig. 5E-G). The treatment with Syk inhibitor abolished the elevated glycolysis in Reelin overexpressing cells, suggesting a critical role of Syk in Reelin-induced myeloma cell glycolysis.

As Akt was activated in Reelin-expressing cells, we examined the contribution of Akt in Reelin-induced MM proliferation. The addition of PI3K inhibitor LY294002 suppressed mTOR and 4E-BP1 phosphorylation and partially inhibited Cyclin D1 upregulation in pCrl-transfected H929 cells. It did not alter the activation of FAK, Syk, and STAT3 (Fig. 5C). Myeloma cell proliferation was also down-regulated by LY294002 (Fig. 5D). But the difference between pCrl- and vector-transfected cells was not completely abolished (Fig. 5D). Similar results were obtained from U266 cells ( sFig. 3B,C), suggesting that PI3K/Akt/mTOR pathway partially contributes to Reelin-induced myeloma cell proliferation.

**Figure 6:**
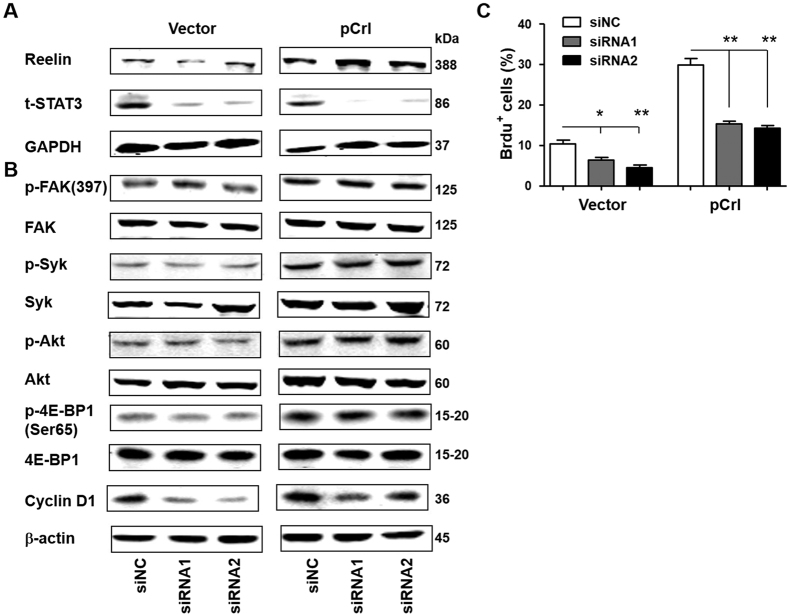
Reelin promotes MM cell growth via STAT3 pathway. (**A**) The knockdown of STAT3 by specific siRNAs. H929 cells were co-transfected with pCrl and STAT3-specific siRNAs (or control siRNA, siNC) or pcDNA3 with siRNAs. Forty hours later, the cells were harvested and cell lysates were subjected to western blotting with Reelin and STAT3-specific antibodies. An antibody specific for GAPDH was used as loading control. (**B,C**) STAT3 contributes to Reelin-induced myeloma cell proliferation. The Reelin and STAT3 siRNAs co-transfected cells were cultured in FN-coated plates. Twenty-four hours later, a fraction of cells were subjected to western blotting with phospho-FAK (Tyr397), FAK, phospho-Syk (Tyr525/526), Syk, phospho-Akt (Ser473), Akt, phospho-4E-BP1 (Ser65), 4E-BP1 and Cyclin D1-specific antibodies. An antibody specific for β-actin was used as the loading control (**B**). The rest of the cells were analyzed for cell proliferation by BrdU staining (**C**). Similar experiments were performed for 3 times, and the representative results were shown.

We next examined the role of STAT3 that is also activated by Reelin-induced Syk phosphorylation. As shown in Fig. 6A,B, the co-transfection of pCrl with STAT3-specific siRNAs in H929 reduced the upregulation of Cyclin D1 but did not alter the phosphorylation of FAK, Syk, Akt and 4E-BP1. The co-transfection with STAT3 siRNA also suppressed Reelin-induced H929 cell growth when compared to control siRNA (Fig. 6C). The transfection of STAT3 siRNA in U266 cells gives similar results ( sFig. 4), suggesting that STAT3 activation contributes to Reelin-induced myeloma cell proliferation.

### Reelin enhances glycolysis through STAT3- and PI3K/Akt/mTOR-regulated HIF1α expression

**Figure 7:**
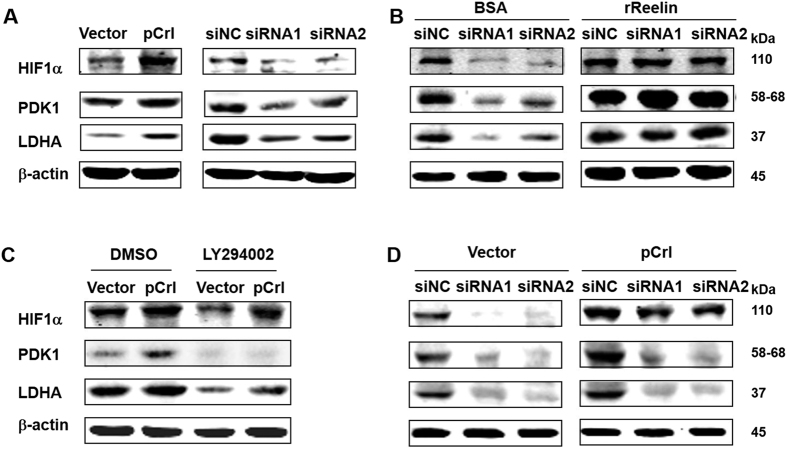
Reelin induced HIF1α upregulation through STAT3 and PI3K/Akt pathways. (**A**) Reelin alters the protein levels of HIF1α and its targets. H929 cells were transfected with pCrl/vector or Reelin specific siRNAs/siNC for 40 hours. The cells were then cultured in FN-coated plates for 24 hours and were subjected to western blotting with HIF1α, PDK1, LDHA, and β-actin-specific antibodies. (**B**) The addition of Reelin protein abolished HIF1α down-regulation in cells with Reelin knockdown. H929 cells were transfected with Reelin-specific siRNA or control siRNA for 40 hours. The cells were then seeded in FN-coated plates with rReelin or BSA control. Twenty-four hours later, the cells were harvested and the cell lysates were subjected to western blotting. (**C**) PI3K/Akt is involved in Reelin-induced HIF1α upregulation. pCrl-transfected H929 cells were treated with DMSO or PI3K inhibitor LY 294002 for 24 hours. The cells were then subjected to western blotting. (**D**) STAT3 is involved in Reelin-induced HIF1α upregulation. H929 cells were co-transfected with pCrl and STAT3-specific siRNAs (or siNC) or pcDNA3 with siRNAs. The cells were then seeded in FN-coated plates for 24 hours and were subjected to western blotting.

The oncogenic roles of STAT3 are highly dependent on one of its transcriptional targets, hypoxia-inducible factor (HIF)^25^,^26^,^27^. The PI3K/Akt/mTOR pathway also upregulates HIF1 level^28^. In addition, it is widely accepted that HIF1α functions as a master regulator in the integration of pathways involved in glycolysis and cell proliferation^29^. Thus, we examined whether Reelin alters HIF1α level. Compared to controls, the protein level of HIF1α under normoxic conditions was increased in H929 cells with Reelin overexpression and was decreased in cells with Reelin knockdown (Fig. 7A). When rReelin protein was given to cells, the HIF1α reduction in Reelin siRNA-transfected cells was abolished (Fig. 7B). We also measured the protein expression of two direct targets of HIF1α, lactate dehydrogenase A (LDHA), and pyruvate dehydrogenase kinase 1 (PDK1). Both targets were upregulated in cells with Reelin overexpression and down-regulated in cells with Reelin knockdown (Fig. 7A). The addition of rReelin abolished Reelin siRNA-induced down-regulation of LDHA and PDK1 (Fig. 7B). We further examined the expression pattern of these genes in MM patients in GSE24080 (sTable 2). The group with high RELN expression had higher mRNA levels of LDHA (P = 0.036), PDK1 (P < 0.001), HIF1A (P = 0.023), and CCND1 (P values for two CCND1 probes were 0.018 and 0.01, respectively). These results indicate that Reelin induces the upregulation of HIF1α and its target genes LDHA and PDK1 in myeloma cells.

We also investigated the roles of PI3K/Akt and STAT3 on the cellular levels of HIF1α and its targets. The treatment with PI3K inhibitor partially reduced the elevation of HIF1α, LDHA, and PDK1 in Reelin-overexpressing cells (Fig. 7C). When H929 cells were co-transfected with pCrl and STAT3 siRNAs, the increase in HIF1α, LDHA, and PDK1 proteins was abolished (Fig. 7D and sFig. 5). These results suggest that Reelin-induced PI3k/Akt and STAT3 activation contributes to the elevation of HIF1α protein and subsequent glycolysis in myeloma cells.

## Discussion

The extracellular matrix protein Reelin has been found to promote the proliferation of intestinal and submandibular gland epithelial cells and granulose cells via ApoERs (VLDLR)/Dab1/PI3K/Akt and MAPK pathways^30^,^31^,^32^. Whether similar function and similar signaling pathways can be found in Reelin-expressing tumors are not clear. In this study, we demonstrate that Reelin is negatively associated with myeloma prognosis and plays an important role in the regulation of cancer cell growth both *in vitro and in vivo*. The FAK/Syk/Akt/mTOR and STAT3 signaling pathways activated by Reelin significantly contribute to the increase in myeloma cell proliferation and glycolysis. It indicates that Reelin may serve as an excellent therapeutic target for myeloma treatment.

The interaction of extracellular matrix macromolecules and their cell surface receptors, in particular, integrins, contribute to cell proliferation in physiological and pathological conditions^33^,^34^,^35^. This is realized by the cross-talk between integrins and growth factor receptors, regulatory loop between integrin/FAK and tumor suppressor p53, and integrin-dependent signaling pathways such as PI3K/Akt/mTOR, NF-κB, and MEK/ERK. Recently, it was also found that β2 integrin could activate Syk/STAT3/5 signaling pathway and induce cell division in acute myeloid leukemia cells^36^. Whether the similar pathway is involved in the proliferation of normal or malignant B cells is not completely clear although suppression of Syk activity was found to reduce myeloma cell proliferation^37^,^38^,^39^. Our investigation on multiple myeloma cell lines indicates that Reelin-induced activation of integrin β1^10^ and its downstream FAK/Syk results in the phosphorylation of both PI3K/Akt/mTOR and STAT3, providing combinatorial cues for myeloma cell proliferation. Signaling through IL-6 and ERK is probably not involved^10^. In addition, the blockage of Reelin secretion by Brefeldin A could diminish Reelin-induced integrin β1 activation in myeloma cells^10^. Together with the fact that reduced cell proliferation was found in myeloma cells with Reelin knockdown, these suggest that Reelin has an autocrine effect and is required to be released from the cells to promote integrin β1 activation and cell growth.

The activation of STAT3 drives the transcription of multiple cell-cycle-related genes, such as CCND1. The activation of PI3K/Akt/mTOR stimulates cap-dependent translation of these mRNAs through the phosphorylation of p70S6K and 4E-BP1, two downstream targets of mTOR. The knockdown of STAT3 and suppression of PI3K each significantly decreased but did not completely abolish Reelin-mediated Cyclin D1 upreguation and myeloma cell proliferation. Thus, Reelin-induced phosphorylation of Akt and STAT3 may act in concerto to promote myeloma cell growth.

Warburg effect provides cancer cells with a unique mechanism to produce ATP for survival, chemoresistance, and proliferation^40^. Whether Reelin/integrin β1/FAK pathway contributes to the Warburg effect in cancer cells is not known. Wang, *et al*. recently reported that integrin β5/FAK activation could rescue cisplatin-induced glycolysis inhibition in breast and cervical cancer cell lines, implicating that integrin may be involved in metabolic reprogramming^41^. However, the detailed signaling pathways that connect integrin β5/FAK activation and glycolysis-related gene upregulation were not investigated. Our results demonstrated in HMCLs that integrin β1, activated by Reelin, directly upregulated glycolysis. The activation of Syk and its downstream Akt/STAT3 pathways contributed to this enhanced glycolysis.

It has been well identified that both PI3K/Akt/mTOR and STAT3 are the key players associated with the Warburg effect^25^,^26^,^27^,^28^,^42^,^43^,^44^. These pathways upregulate or activate the center molecule HIF1α which directly or indirectly regulate the expression of genes that code for glycolytic enzymes and enzymes that convert glucose into lactate, leading to an increase in aerobic glycolysis^45^,^46^,^47^. Recently, it was shown that the inhibition of HIF1α and LDHA specifically suppressed myeloma cell growth and restored the sensitivity of drug resistant cell lines^48^. Our results demonstrated that the increase in glycolysis and the upregulation of HIF1α and its targets, LDHA and PDK1, can be induced by an extracellular matrix protein Reelin through its activation of FAK/Syk and the subsequent activation of PI3K/Akt/mTOR and STAT3 pathways. The Akt and STAT3 likely work together to promote HIF1α and its target gene upregulation. Reelin’s role in myeloma cell glycolysis was further supported by the clinical data from GSE24080 as high RELN-expressing patients also revealed high levels of HIF1α, PDK1, and LDHA expression.

Resistance to chemotherapeutic agents is one of the major obstacles to the successful treatment of multiple myeloma. Accumulating studies have shown that chemoresistance is closely associated with enhanced glucose metabolism in a variety of cancer types, including myeloma^49^. Defective mitochondrial ATP production, elevated aerobic glycolysis, and enhanced HIF1α-mediated signaling are major characteristics of these drug-resistant tumor cells^50^,^51^. Knockdown or specific inhibition of HIF1α restores the sensitivity of tumor cells to chemotherapeutic drugs^48^,^52^. We previously demonstrated that Reelin-overexpressing MM cells had increased resistance to multiple chemotherapeutic drugs including cisplatin, bortezomib, imatinib mesylate, and Doxorubicin^10^. The current study revealed that Reelin/FAK/Syk/Akt and STAT3 pathways in myeloma cells promoted HIF1α expression and cell glycolysis. It is thus likely that the contribution of Reelin in myeloma chemoresistance is at least partially through enhanced glucose metabolism.

Taken together, the current study and our previous one demonstrate that high expression of Reelin in myeloma patients is associated with tumor progression and poor patient outcome^10^. Reelin contributes to cancer progression by activating integrin β1/Syk/Akt and STAT3 pathways and promoting cell adhesion, survival, proliferation, and drug resistance. Our results further reveal a novel biologic role of Reelin in HIF1α expression and cellular glucose metabolism. It provides an opportunity for myeloma therapeutic intervention by inhibiting Reelin and its integrin pathway.

## Materials and Methods

### GEO dataset and clinical information of MM patients

The gene microarray expression data and corresponding clinical information of 565 newly diagnosed MM patients were obtained from publicly available GEO database (GSE24080 (Affymetrix HG-U133_Plus_2.0 array) (www.ncbi.nlm.nih.gov/geo/query/acc.cgi?acc=GSE24080)^15^.

### Human myeloma cell lines and cell culture

The human myeloma cell line NCI-H929 (shown as H929) and U266 were kindly provided by Prof. Jian Hou from Shanghai Chang Zheng Hospital and Prof. Yu Zhang from Peking University Health Science Center (Beijing, China), respectively. The cells were cultured in RPMI 1640 (GibCo, Life Technologies, Grand Island, NY, USA) supplemented with 10% fetal bovine serum (Hyclone, ThermoFisher Scientific, Waltham, MA, USA), 2 mmol/L glutamine, and 1% penicillin/streptomycin (GibCo). The cells were cultured at 37 °C in a humidified atmosphere of 5% CO_2_.

The 96-well plate was coated with 40 μg/ml of FN (Sigma) or 5% bovine serum albumin (BSA) in PBS at 37 °C for 1 hour. BSA in PBS (1%) was then used to block nonspecific binding sites in the wells at 37 °C for 1 h before the experiment. Transfected HMCLs were then added to FN- or BSA-coated 96-well plates at 37 °C for 24 hours. In some experiments, recombinant Reelin protein (1 μg/ml), Syk inhibitor IV, BAY 61–3606 (1 μmol/L, Merck Millipore), or PI3K inhibitor LY 294002 (50 μmol/L, Cell Signaling Technology) were added in HMCLs in plates.

Flow cytometric analysis was performed using a FACS Gallios (Beckman Coulter, Indianapolis, IN, USA). Anti-AnnexinV-FITC, anti-AnnexinV-APC, anti-Ki67-PE antibodies were purchased from BD PharMingen (San Diego, CA, USA). Brdu Flow Kit was purchased from BD PharMingen and the experiments were performed according to the manufacturer’s instructions. Cell counting kit-8 (CCK8) assays (DOJINDO Molecular Technologies, Minato-ku, Tokyo, Japan) were performed according to the manufacturer’s instructions.

### Quantitative RT-PCR

Total RNA was isolated from the indicated cells using TRIzol (Invitrogen, Grand Island, NY, USA), according to the manufacturer’s instructions. The cDNA was synthesized using the Transcriptor First Strand cDNA Synthesis kit (Tiangen, Beijing, China). Real-time PCR was prepared using a PCR Master Mix (Roche, Basel Schweiz, Switzerland) according to the manufacturer’s protocol. It was then performed on an iCycler (Bio-Rad Laboratories, Hercules, CA, USA). The primer sequences are shown in sTable 3. The PCR conditions were 94 °C for 12 s, 60/58 °C for 12 s, and 72 °C for 12 s. The quantification was based on ∆∆CT calculations and was normalized to GAPDH.

### Transient and stable transfection

The full-length Reelin-expressing vector, pCrl, was a generous gift from Prof. Tom Curran (The Children’s Hospital of Philadelphia, Philadelphia, PA). pcDNA3 was used as a control vector. siRNAs against *RELN* and *STAT3* were purchased from RIBOBIO (Guangzhou, China). MM cells growing at logarithmic phase were transfected with 10 μg pCrl or control vector pcDNA3, or 300 pmol Reelin-specific siRNA, or negative control siRNA (siNC) using electroporation (Multiporator, Eppendorf, Hamburg, Germany). The sequences of siRNAs were shown in sTable 4.

H929 cells transfected with pCrl or pcDNA3 were cultured in the presence of 400 μg/ml of G418. The cell clone stably expressing highest level of Reelin was selected for animal experiments.

### Plasmacytoma xenograft mouse model

Eight-week old female non-obese diabetic (NOD)/severe combined immunodeficient (SCID) mice were purchased from Weitonglihua (Beijing, China). The mice were kept in a specific pathogen-free facility at Peking University Health Science Center (Beijing, China). The experimental procedures on use and care of animals had been approved by the Institutional Animal Care and Use Committee of Peking University Health Science Center. This study was carried out in accordance with these approved guidelines. The mice (6 in each group) were subcutaneously inoculated with vector- or pCrl-stably transfected H929 cells (1 × 10^7^) in 100 μL of serum-free RPMI-1640. When palpable tumors were developed (about 2 weeks post-inoculation, Day 0), the tumors were measured with a caliper once every 3 days to estimate the tumor volume. The following formula was used: V = 0.5 × a × b^2^, where “a” and “b” were the long and short diameters of the tumor, respectively. The mice were sacrificed at Day 24 or when the tumors reached 2 cm in diameter to prevent unnecessary suffering.

Excised tumors from mice were immediately fixed and stored in 4% buffered formaldehyde. The fixed tissues were delivered to Goodbio Technology Company (Wuhan, China) for dehydration and paraffin embedding. Hematoxylin and eosin (H&E) staining on the paraffin sections was performed by Goodbio Technology Company. For Ki67 staining, the sections were antigen retrieved by heating for 2 min in 10 mM citric acid (pH 6.0) and stained with polyclonal rabbit anti-Ki67 (Abcam; 2 μg/ml). The pictures were taken with an Olympus microscope (Center Valley, PA, USA).

### Immunoblotting

After cell culture, HMCLs were harvested and washed twice with ice-cold PBS. To achieve whole-cell lysates, the cells were incubated for 10 minutes at 4 °C in Triton X-100 lysis buffer (30 mM Tris-HCl pH7.5, 150 mM NaCl, 25 mM NaF, 1% Triton X-100, 10% glycerol, 2 mM Sodium orthovanadate). These lysates were subjected to 6–10% gradient polyacrylamide gels and transferred to nitrocellulose membrane (Whatman, GE Healthcare Life Sciences, Pittsburgh, PA, USA). The primary antibodies used were anti-Reelin, purchased from Abcam (Cambridge, MA, USA), anti-phospho-FAK (Tyr397), anti-FAK, anti-phospho-STAT3 (Tyr705), anti-STAT3, anti-phospho-Syk (Tyr525/526), anti-Syk, anti-phospho-Akt (Ser473), anti-Akt, anti-phospho-mTOR (Ser2448), anti-mTOR, anti-phospho-4E-BP1 (Ser65), anti-4E-BP1, anti-phospho-Rb (Ser780), anti-Rb, anti-HIF1α, anti-PDK1, anti-LDHA, anti-Cyclin D1, anti-β-Actin and anti-GAPDH from Cell Signaling Technology (Danvers, MA, USA). Goat-anti-rabbit IRDye 800CW, Goat-anti-mouse IRDye 800CW (LI-COR Biosciences, Lincoln, NE, USA), anti-mouse IgG HRP conjugate, anti-rabbit IgG HRP conjugate (Promega, Madison, WI, USA) were used as the secondary antibodies. The immunoreactive bands were detected by fluorescence with LiCor Odyssey Gel imaging Scanner, or chemiluminescence with ECL detection reagents (ThermoFisher Scientific) and exposed to ImageQuant^TM^ LAS 500 (GE Healthcare Life Sciences).

### Glycolysis measurements

Transfected cells in fresh RPMI-1640 (serum-free for LDH measurement and 10% fetal bovine serum for L-Lactate measurement) were cultured in FN-coated 96-well plate for 24 hours in the presence or absence of Syk inhibitor, BAY 61-3606 (1 μmol/L, Merck Millipore). The conditioned medium in each well was then collected. The amount of LDH in the medium was determined using the Lactate Assay Kit and the level of L-Lactate was determined using the L-Lactate Assay Kit (Abcam, Cambridge, MA, USA). The glucose uptake by myeloma cells were measured using the Glucose Uptake Assay Kit (Abcam). Briefly, the transfected cells were resuspended in serum-free RPMI-1640 and seeded in FN-coated 96-well plate in the presence or absence of Syk inhibitor. After 24 hours, the cells were washed twice with PBS and were starved for glucose using 100 μl 2% BSA buffer for 40-minute of pre-incubation. Each well was then added 10 μl of 10 mM 2-deoxy-d-glucose (2-DG) and the cells were incubated for 20 minutes. The cells were then washed for 3 times with PBS to remove exogenous 2-DG. The unused NAD(P) left in the cells was degraded by extraction buffer and samples were neutralized by neutralization buffer. After spin, the supernatant was collected for glucose uptake measurement.

### Statistical analysis

Chi-square test, logistic regression and *t*-test were used to compare the demographic characteristics of patients. The relative expression levels of RELN from myeloma patients were first transformed by log-base 2 and were then analyzed by a hierarchical cluster analysis with Ward’s method. The cut-off value (810) was defined. The Kaplan-Meier method was used to plot the OS and EFS, which were compared between patients with high and low *RELN* expression using the log-rank test. Hazard ratios (HRs) and 95% CIs were generated, with a HR < 1.0 indicating survival benefit (or reduced mortality). Cox regression method was used for multivariate analysis of OS and EFS. The data from HMCLs were evaluated by two-tailed Student’s *t*-test and 2-way ANOVA. All data are presented as mean ± SD. The calculations were performed using Graphpad and SPSS 20.0 (SPSS Inc, Chicago, IL). The following terminology is used to denote the statistical significance: *p < 0.05, **p < 0.01, ***p < 0.005, ns, not statistically significant.

## Additional Information

**How to cite this article**: Qin, X. *et al*. Extracellular matrix protein Reelin promotes myeloma progression by facilitating tumor cell proliferation and glycolysis. *Sci. Rep.* **7**, 45305; doi: 10.1038/srep45305 (2017).

**Publisher's note:** Springer Nature remains neutral with regard to jurisdictional claims in published maps and institutional affiliations.

## Supplementary Material

Supplementary Figures and Tables
