# Supplementary material for: Extracellular matrix protein Reelin promotes myeloma progression by facilitating tumor cell proliferation and glycolysis
Source: Sci Rep. 2017 Mar 27;7:45305. doi: 10.1038/srep45305 (PMC5366887; doi:10.1038/srep45305)
Supplement: Supplementary Figures and Tables [file srep45305-s1.doc]

**Supplementary information**

Extracellular matrix protein Reelin promotes myeloma progression by facilitating tumor cell proliferation and glycolysis

Xiaodan Qin^a#^, Liang Lin^a#^, Li Cao^a^, Xinwei Zhang^a^, Xiao Song^a^, Jie Hao^a^, Yan Zhang^a^, Risheng Wei^b^, Xiaojun Huang^c^, Jin Lu^c^*, Qing Ge^a^*

**Supplemental tables**

sTable 1 The relationship between RELN expression and clinical information of newly diagnosed myeloma patients from GSE24080

| Characteristic | RELN | | *P* value |
| --- | --- | --- | --- |
|  | High | Low |  |
| AGE | 57.7 ± 0.61 | 56.6 ± 0.55 | 0.168 |
| SEX |  |  |  |
| male | 136/230 (59%) | 120/308 (39%) | 0.655 |
| female | 94/230 (41%) | 188/308 (61%) |  |
| BMPC (%) | 46.2 ± 1.7 | 46.5 ± 1.6 | 0.127 |
| HGB (g/dl) | 111.6 ± 1.1 | 113.3 ± 1.1 | 0.275 |
| B2M (mg/l) | 5.00 ± 0.40 | 4.44 ± 0.26 | 0.227 |
| LDH (U/l) | 184.6 ± 5.0 | 162.0 ± 3.1 | <0.001 |
| CREAT (mg/dl) | 1.38 ± 0.09 | 1.27 ± 0.07 | 0.332 |
| CRP (mg/l) | 11.49 ± 1.37 | 11.50 ± 1.34 | 0.994 |
| ALB (g/l) | 40.2 ± 0.40 | 40.7 ± 0.32 | 0.350 |
| MRI | 12.6 ± 1.0 | 9.7 ± 0.8 | 0.023 |

AGE, age at registration (years); SEX, sex (male or female); BMPC, bone marrow biopsy plasma cells (%); HGB, Haemoglobin, g/dl; B2M, beta-2 microblobulin, mg/l; LDH, lactate dehydrogenase, U/l; CREAT, creatinine, mg/dl; CRP, C-reactive protein, mg/l; ALB, albumin, g/l; MRI, number of Magnetic Resonance Imaging (MRI)-defined focal lesions (skull, spine, pelvis).

sTable 2 The relationship between the expressions of RELN and selected genes of myeloma patients (GSE24080)

| Characteristic | RELN | | *P* value |
| --- | --- | --- | --- |
|  | High | Low |  |
| LDHA (200650_s_at) | 16080 ± 357 | 15116 ± 294 | 0.036 |
| PDK1 (206686_at) | 2103 ± 80.2 | 1665 ± 64.0 | <0.001 |
| PDK1 (226452_at) | 13840 ± 410 | 11283 ± 340 | <0.001 |
| CCND1 (208711_s_at) | 2442 ± 338 | 3802 ± 377 | 0.010 |
| CCND1 (208712_at) | 2262 ± 272 | 3269 ± 305 | 0.018 |
| HIF1A | 12.6 ± 1.0 | 9.7 ± 0.8 | 0.023 |

sTable 3 Primers used in qRT-PCR analyses (5’-3’)

| RELN | forward | GATGGGCGGCGTCAGCTAAT |
| --- | --- | --- |
|  | reverse | GGCTCTGCACGTGCTCAGAA |
| GAPDH | forward | ACCCACTCCTCCACCTTTGA |
|  | reverse | CTGTTGCTGTAGCCAAATTCGT |
| p21 | forward | AGACCAGCATGACAGATTTC |
|  | reverse | ACTGAGACTAAGGCAGAAGA |
| CCND1 | forward | CAATGACCCCGCACGATTTC |
|  | reverse | CATGGAGGGCGGATTGGAA |
| PIM1 | forward | ACGCTTGCTCTGTTTGTGG |
|  | reverse | CTGGAAGGCACACCATCC |
| c-Myc | forward | AATGAAAAGGCCCCCAAGGTAGTTATCC |
|  | reverse | GTCGTTTCCGCAACAACAAGTCCTCTTC |
| SOX2 | forward | TACAGCATGTCCTACTCGCAG |
|  | reverse | GAGGAAGAGGTAACCACAGGG |
| NANOG | forward | TTTGTGGGCCTGAAGAAAACT |
|  | reverse | AGGGCTGTCCTGAATAAGCAG |
| Oct3/4 | forward | CAAAGCAGAAACCCTCGTGC |
|  | reverse | TCTCACTCGGTTCTCGATACTG |
| IGF-1 | forward | GCTCTTCAGTTCGTGTGTGGA |
|  | reverse | GCCTCCTTAGATCACAGCTCC |
| ICAM | forward | ATGCCCAGACATCTGTGTCC |
|  | reverse | GGGGTCTCTATGCCCAACAA |
| VEGFA | forward | AAGATCCGCAGACGTGTAAATGTT |
|  | reverse | CGGCTTGTCACATCTGCAAGTA |

sTable 4 Sequences of siRNAs.

| siRNAs | Sense strand (5’-3’) |
| --- | --- |
| Reelin siRNA1 | CCAGCAUCAUCGUGUUAUAdTdT |
| Reelin siRNA2 | GGCGAUUGAUAAUGUUGUAdTdT |
| STAT3 siRNA1 | GAGAUUGACCAGCAGUAUAdTdT |
| STAT3 siRNA2 | CCAACAAUCCCAAGAAUGUdTdT |

**Supplemental Figures**


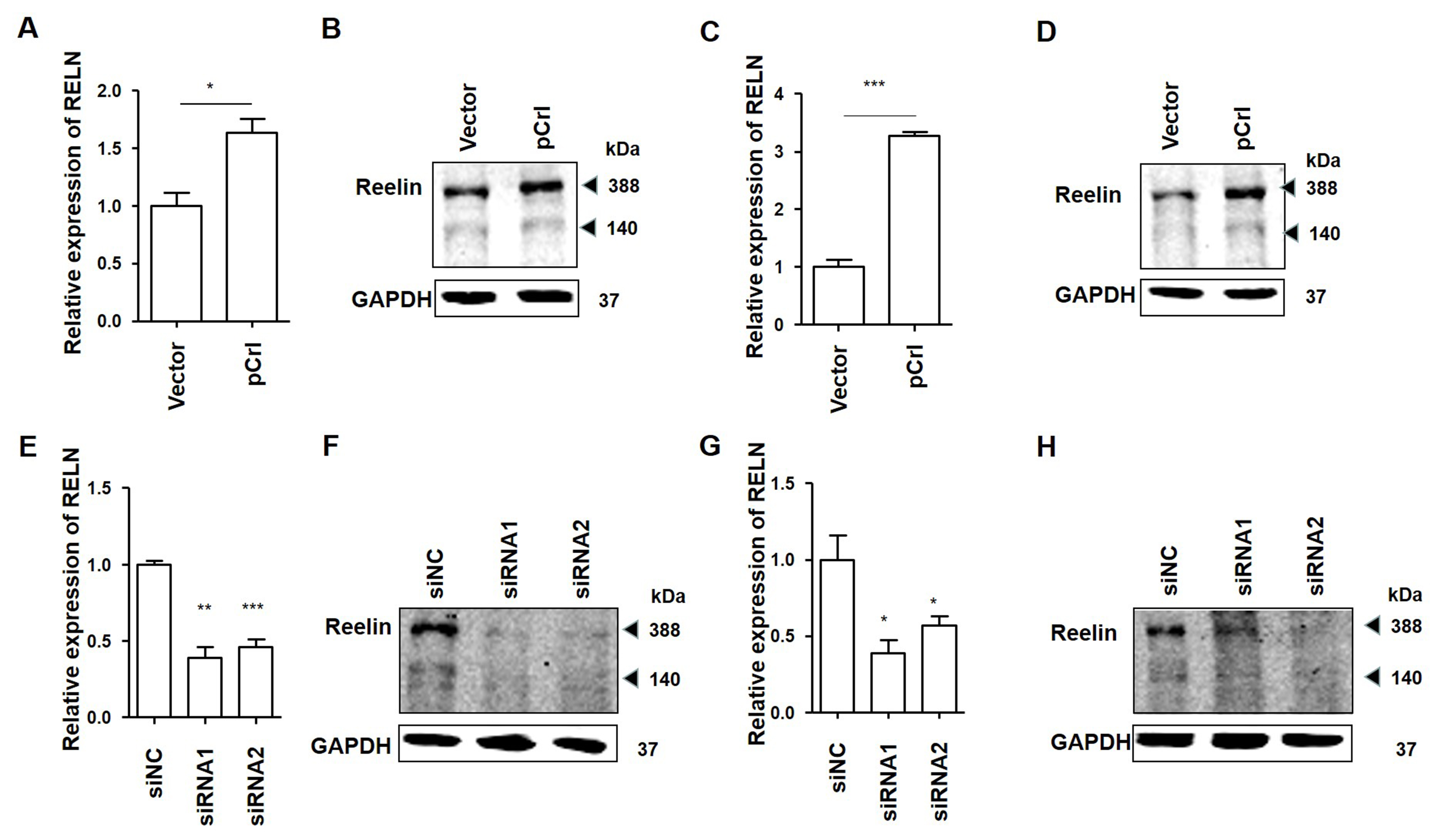


**Supplemental Figure 1** Transfection of Reelin-expressing plasmid and *RELN*-specific siRNAs in HMCLs. (A-B) Overexpression of Reelin in H929 cells. H929 cells were transfected with 10 μg of Reelin-expressing plasmid (pCrl) or control plasmid pcDNA3 (vector). The cells were harvested 24 hours later and the mRNA expression of RELN was analyzed by quantitative RT-PCR (A). The protein level of Reelin was measured by western blotting at forty hours post-transfection (B). Two Reelin immunoreactive bands (full length isoform of 388 KDa and a cleaved fragment of 140 KDa) were revealed with the 388 KDa as the major form of Reelin protein in cell lysates. (C-D) The expression of Reelin mRNA (C) and protein (D) in pCrl-transfected U266 cells. (E-F) Knockdown of Reelin in H929 cells. H929 cells were transfected with 300 pmol Reelin-specific or control (siNC) siRNAs. The cells were harvested 24 or 40 hours later. The mRNA expression of Reelin was analyzed by quantitative RT-PCR (E) and Reelin protein expression was analyzed by western blotting (F). (G-H) The expression of Reelin mRNA (G) and protein (H) in Reelin siRNA-transfected U266 cells.


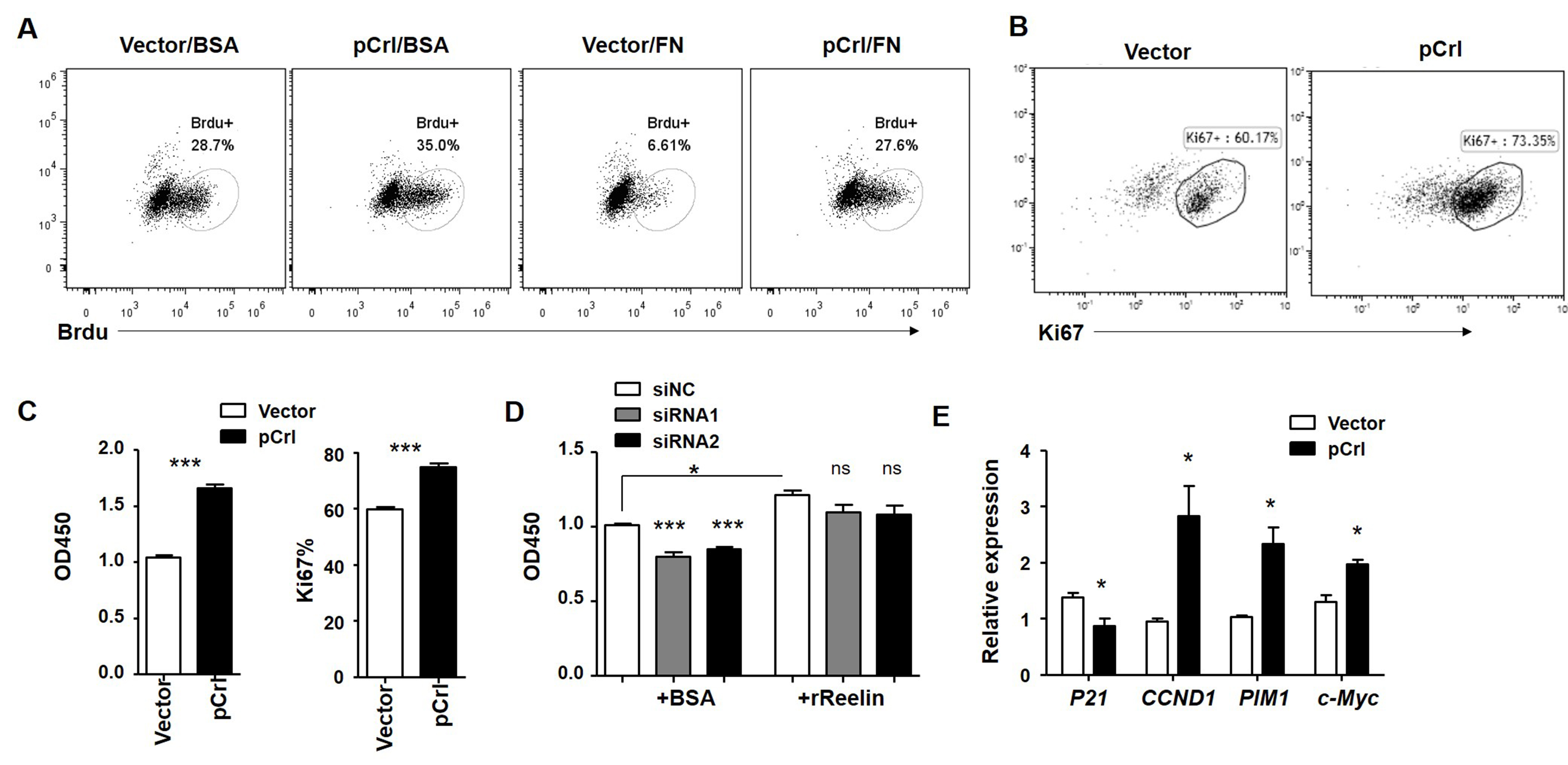


**Supplemental Figure 2** Reelin promotes MM cell proliferation *in vitro*. (A-B) Reelin promotes H929 cell proliferation. H929 cells were transfected with pCrl or vector for 40 hours. The cells were then seeded in 5% BSA- or 40 μg/ml of FN-coated plates. The cell proliferation was analyzed 24 hours later by BrdU (A) and Ki67 staining and flow cytometry. (C) Reelin overexpression promotes U266 cell proliferation. U266 cells were transfected with pCrl or vector for 40 hours. The cells were then seeded in FN-coated plates. The cell proliferation was analyzed by CCK8 method and Ki67 staining at 24 hours later. (D) Reelin knockdown suppresses U266 cell growth. U266 cells were transfected with Reelin specific siRNA or control siRNA (siNC) for 40 hours. The cells were then seeded in FN-coated plates in the presence or absence of rReelin. The cell proliferation was analyzed by CCK8 method at 24 hours later. (E) Reelin alters the expression of proliferation-related genes in U266 cells. The cells in (C) were extracted for total RNAs and the gene expression was analyzed by quantitative RT-PCR. The experiments were performed for three times and similar results were obtained.


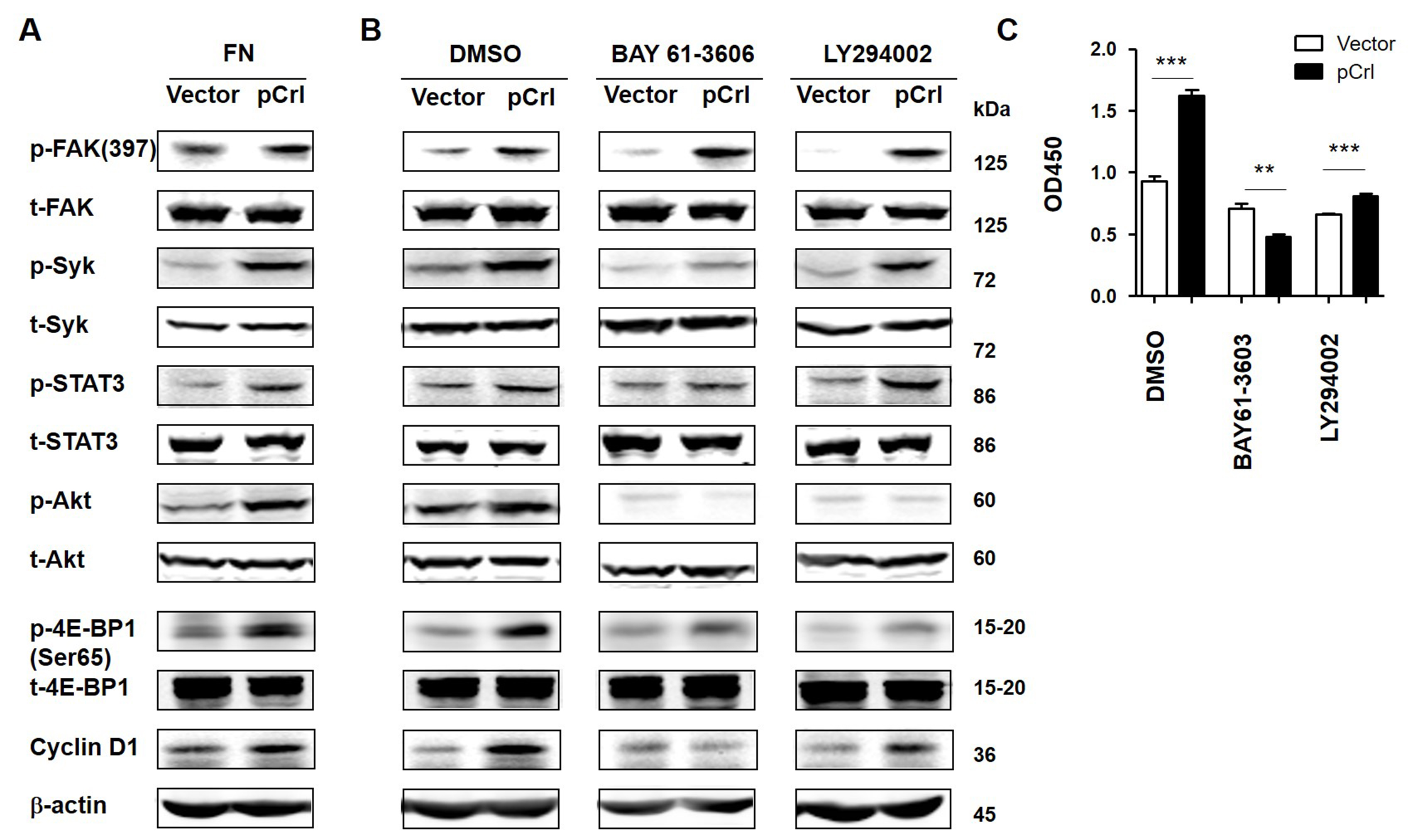


**Supplemental Figure 3** Reelin promotes MM cell growth via Syk and Akt pathways. (A) U266 cells were transfected with pCrl or vector for 40 hours. The cells were then seeded in FN-coated plates. One hour later, the cells were harvested and cell lysates were subjected to western blotting with phospho-FAK (Tyr397), FAK, phospho-STAT3 (Tyr705), STAT3, phospho-Syk (Tyr525/526), Syk, phospho-Akt (Ser473), Akt, phospho-mTOR (Ser2448)，mTOR, phospho-4E-BP1 (Ser65)，4E-BP1 and Cyclin D1-specific antibodies. An antibody specific for β-actin was used as the loading control. (B-C) The suppression effect of Syk and PI3K inhibitors on Reelin-mediated cell growth. U266 cells were transfected with pCrl or control plasmid and were then cultured in FN-coated plates. The cells were treated with DMSO, Syk inhibitor BAY 61-3606, or PI3K inhibitor LY 294002 for 24 hours. A fraction of the cells were lysed and subjected to western blotting (B) and the rest were measured by CCK8 method (C). The results are representative of two to three independent experiments.


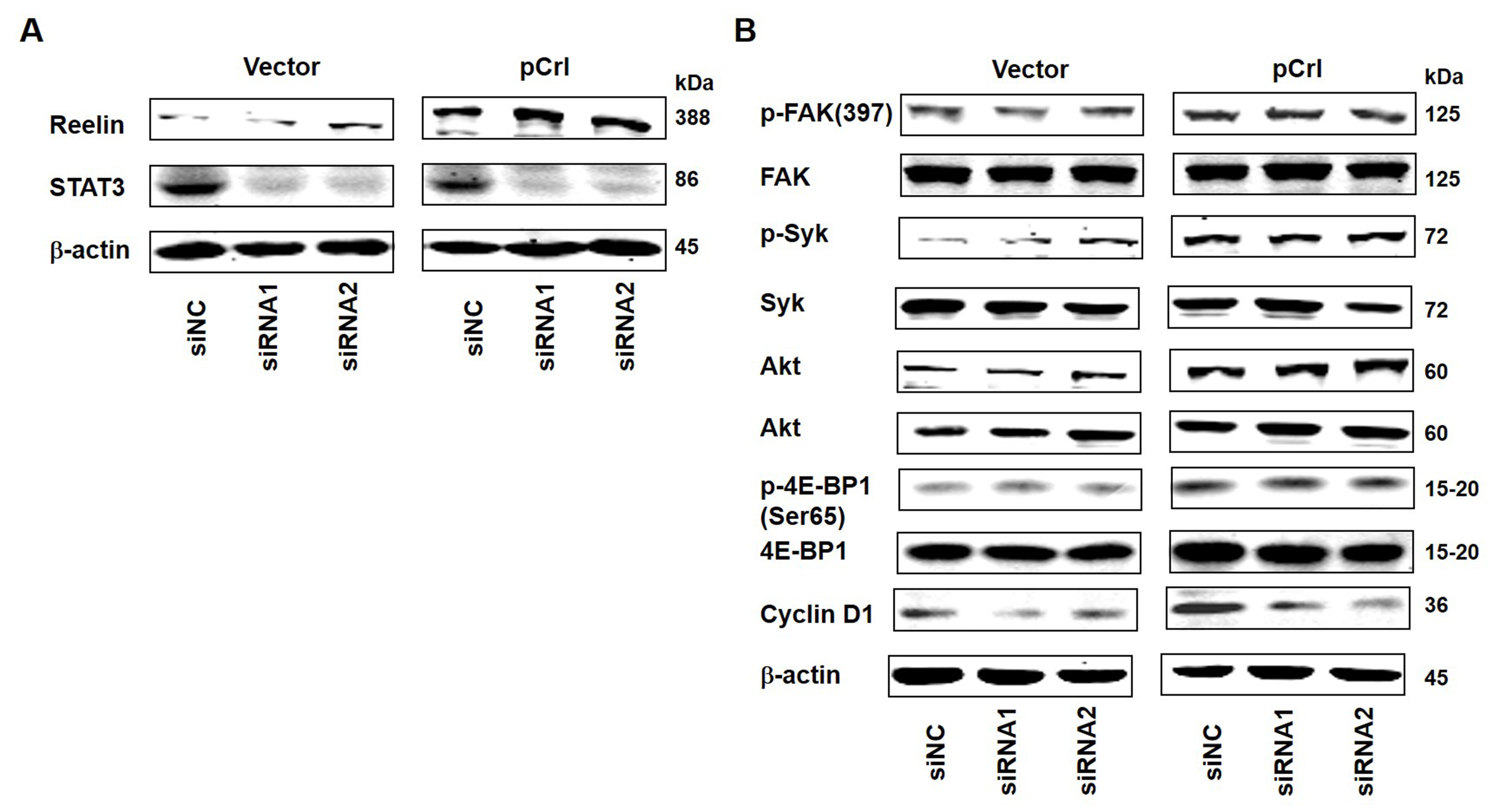


**Supplemental Figure 4** Reelin promotes MM cell growth via STAT3 pathway. (A) The knockdown of STAT3 in U266 cells. U266 cells were co-transfected with pCrl and STAT3-specific siRNAs (or siNC) or pcDNA3 with siRNAs. Forty hours later, the cells were harvested and cell lysates were subjected to western blotting with Reelin- and STAT3-specific antibodies. An antibody specific for GAPDH was used as loading control. (B) STAT3 contributes to Reelin-induced myeloma cell growth. The pCrl and STAT3 siRNA co-transfected cells were cultured in FN-coated plates. A fraction of the cells were harvested and cell lysates were subjected to western blotting with phospho-FAK (Tyr397), FAK, phospho-Syk (Tyr525/526), Syk, phospho-Akt (Ser473), Akt, phospho-4E-BP1 (Ser65), 4E-BP1 and Cyclin D1-specific antibodies. An antibody specific for β-actin was used as the loading control. The experiments were repeated for two times.


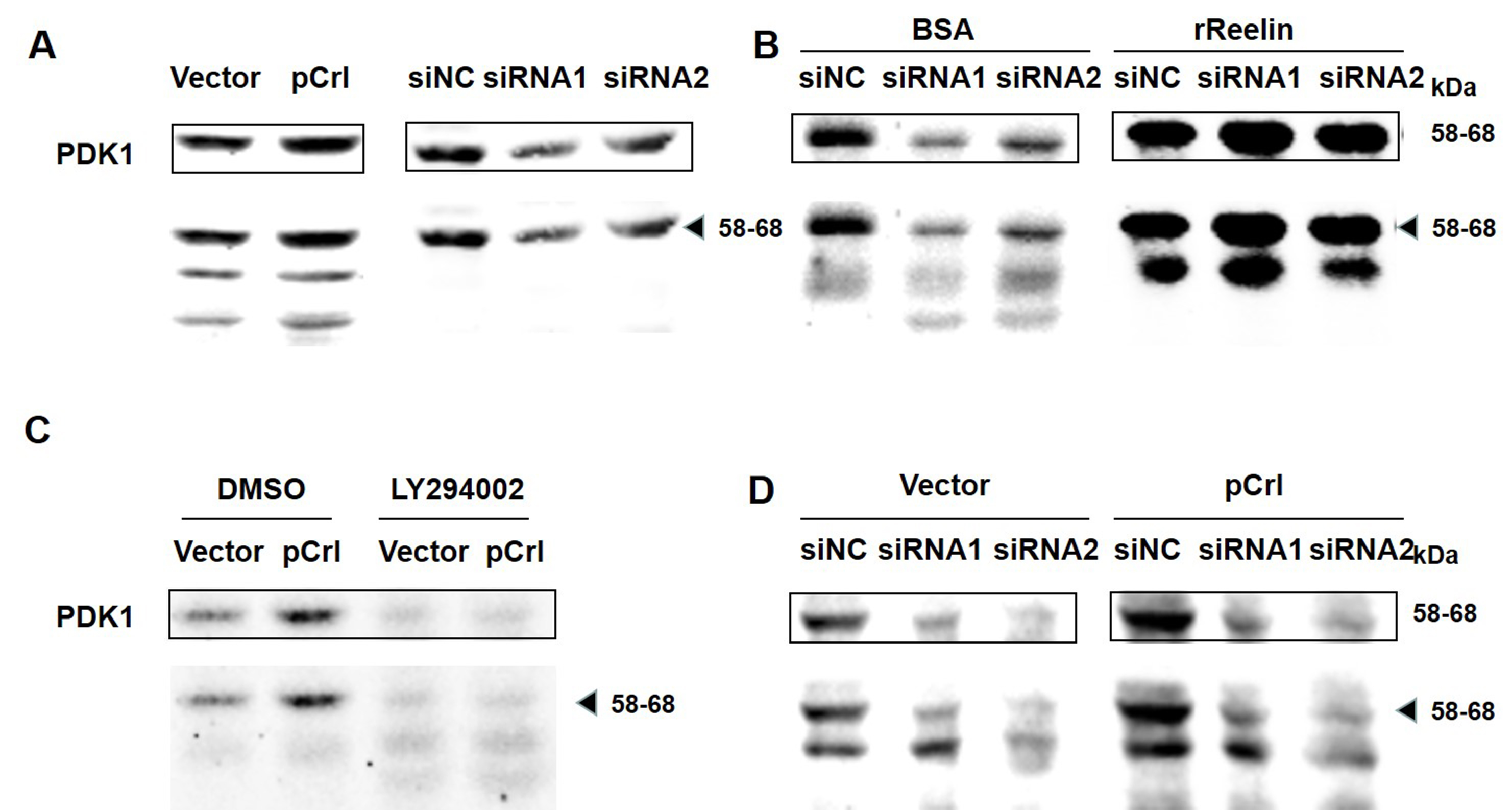


Supplemental Figure 5 Enlarged western blotting of PDK1 for Figure 7. The bands in black box were cut ones with band size of 56-68 kDa. (A) Reelin alters the protein levels of PDK1. H929 cells were transfected with pCrl/vector or Reelin specific siRNAs/siNC for 40 hours. The cells were then cultured in FN-coated plates for 24 hours and were subjected to western blotting with PDK1-specific antibodies. (B) The addition of Reelin protein abolished PDK1 down-regulation in cells with Reelin knockdown. H929 cells were transfected with Reelin-specific siRNA or control siRNA for 40 hours. The cells were then seeded in FN-coated plates with rReelin or BSA control. Twenty-four hours later, the cells were harvested and the cell lysates were subjected to western blotting. (C) PI3K/Akt is involved in Reelin-induced PDK1 upregulation. pCrl-transfected H929 cells were treated with DMSO or PI3K inhibitor LY 294002 for 24 hours. The cells were then subjected to western blotting. (D) STAT3 is involved in Reelin-induced PDK1 upregulation. H929 cells were co-transfected with pCrl and STAT3-specific siRNAs (or siNC) or pcDNA3 with siRNAs. The cells were then seeded in FN-coated plates for 24 hours and were subjected to western blotting.
